# Factors Associated With Length of Hospital Stay for Forensic Psychiatric Inpatients With Intellectual Disabilities

**DOI:** 10.1111/jar.70065

**Published:** 2025-05-08

**Authors:** Penelope McKenna, Rosie England, Carmen Fadzelmulla‐Moreno, Paul A. Thompson, Harm Boer, Peter E. Langdon

**Affiliations:** ^1^ Brooklands Hospital Coventry and Warwickshire Partnership NHS Trust UK; ^2^ Centre for Research in Intellectual and Developmental Disabilities (CIDD) University of Warwick Coventry UK; ^3^ Intellectual Disabilities Research Institute (IDRIS) University of Birmingham UK; ^4^ Learning Disabilities and Autism Services West Midlands UK; ^5^ Herefordshire and Worcestershire Health and Care NHS Trust West Midlands Partnership Alliance UK; ^6^ Birmingham Community Healthcare NHS Foundation Trust Birmingham UK

**Keywords:** forensic mental health, hospital admission, learning disabilities, neurodevelopmental disorders, psychiatry

## Abstract

**Introduction:**

The aim of this study was to examine factors associated with length of stay within a psychiatric hospital for patients with intellectual disabilities who have a forensic history.

**Methods:**

Data about 111 patients were gathered retrospectively from historical records for the period of February 2011 to March 2021. Negative binomial regression was then used to examine the relationship between selected predictor variables and length of stay.

**Results:**

Patients who were older upon admission and those who had received psychological therapies or positive behavioural support (PBS) had a significantly longer length of stay. Those with a diagnosis of a neurodevelopmental disorder had a significantly shorter length of stay. All other predictors were not statistically significant.

**Conclusions:**

There was evidence of a clinical improvement at discharge and those with autism or ADHD had a shorter length of stay. Similar studies with larger sample sizes should be completed across England.

## 1 Introduction

It is estimated that approximately 1.9% of the population of the United Kingdom has a diagnosed intellectual disability and this number appears to be increasing (Public Health England 2018). An intellectual disability is characterised by difficulties with intellectual and adaptive functioning which begins during childhood (Hughes‐McCormack et al. 2017). This population also has a rate of mental illness that is around seven times higher than the general population (Public Health England 2016; Vereenooghe et al. 2018) and potentially a higher need for inpatient treatment for some individuals (Melvin et al. 2022).

In the United Kingdom, when people with intellectual disabilities or those with mental illness engage in behaviour considered criminal, or are convicted of a crime, diversion to hospital or community settings has been considered appropriate for many years (Reed 1992). For those diverted to hospital in England and Wales, they are usually detained under the Mental Health Act, 1983; however, not all those admitted to secure hospitals will have been convicted of a crime as some with intellectual disabilities are admitted due to the nature or degree of their challenging behaviour (Völlm et al. 2018). The key aim of secure hospitals is to treat mental disorder, rehabilitate and discharge patients back into the community while reducing recidivism and protecting the public (Völlm et al. 2018); there is some evidence to support that this does happen for people with intellectual disabilities (Melvin et al. 2022).

However, concerns have been raised that some will stay in secure hospitals for longer than necessary and the duration of a hospital stay can exceed a prison sentence received for the same offence (Trebilcock and Weaver 2012). This can be extremely costly and raise ethical issues for those within the services being subjected to an overly restrictive environment (Reed 1997; Drummond et al. 2005; Stewart et al. 2013). However, NHS England (2015a, 2015b, 2017) launched the Transforming Care programme, which explicitly aimed to reduce the number of psychiatric beds for people with intellectual disabilities in England. While the Transforming Care programme (NHS England 2015a, 2015b, 2017) was focused upon closing psychiatric beds, while also working to reduce length of stay by promoting timely discharge, the overall objectives of the programme were not achieved (Langdon et al. 2023). Concerns were raised about the Transforming Care programme potentially leading to more people with intellectual disabilities being sent to prison in England, and there is evidence that closing NHS intellectual disability inpatient beds is associated with an increase in the number of prisoners in England in the future (Wild et al. 2022). The Transforming Care programme was replaced in 2019 with a goal to reduce the number of inpatients by less than half of the number within hospital within the year 2015 (Department of Health and Social Care 2019) which was not achieved, and most recently, the government set a target to reduce the number of inpatients by 10% within the years 2025–2026 (NHS England 2025).

Previous research has shown that inpatients with intellectual disabilities often have longer admissions to psychiatric inpatient units than those without intellectual disabilities, most likely due to complexity, but admission is associated with clinical improvements (Abraham et al. 2021; Melvin et al. 2022). While some of this evidence comes from studies about admissions to non‐forensic inpatient services (Abraham et al. 2021), we also know that admission to forensic inpatient services is associated with clinical improvements, but a longer length of stay, relative to those admitted to non‐specialist or specialist non‐forensic inpatient services (Melvin et al. 2022). Those admitted to forensic inpatient services tend to be male, younger, with personality disorder and have a longer length of stay, again due to complexity and risk (Lunsky et al. 2011; Alexander et al. 2010, 2012).

Within the United Kingdom, there are state‐funded specialist forensic and non‐forensic inpatient psychiatric services specifically for those with intellectual disabilities where care is provided by clinicians with expertise in working with this population, which is infrequently offered in other countries (Melvin et al. 2022). The lack of specialist inpatient services for people with intellectual disabilities in other countries has been shown to be related to more clinical symptoms and more psychotropic medication within Canada (White et al. 2010) and higher observation and staffing levels within New York (Lohrer et al. 2002). In the United Kingdom, there is evidence that people with intellectual disabilities within non‐specialist forensic inpatient services are more frequently secluded for longer periods than those within specialist forensic inpatient services (Turner and Mooney 2016).

It is the case that reducing the length of stay within inpatient psychiatric hospitals by improving the quality of care and improving clinical outcomes would be beneficial for people with intellectual disabilities. It is important that we understand which factors predict treatment outcome for inpatients with intellectual disabilities to help refine and improve care pathways. In view of the continuing drive within England to reduce the number of patients within intellectual disabilities or autism admitted to psychiatric hospitals, we aimed to determine whether a set of selected factors was associated with length of stay within a specialist forensic psychiatric hospital for people with intellectual disabilities. To achieve this, we retrospectively gathered data about all patients who were admitted to three secure units within a single hospital in England over a 10‐year period starting in February 2011.

## 2 Methods

### 2.1 Participants

The data was captured from patient records held by Brooklands Hospital, Coventry and Warwickshire Partnership NHS Trust. Only patients with a diagnosis of an intellectual disability are admitted to Brooklands Hospital, which would have been confirmed by an appropriately qualified professional (e.g., psychiatrist, clinical psychologist). Brooklands Hospital is a specialist intellectual disability hospital providing inpatient assessment and treatment services to adults and children with intellectual disabilities. It is comprised of multiple units including a 15‐bed male medium secure unit, a 15‐bed male low secure unit and a 15‐bed female low secure unit specifically for patients with a diagnosis of an intellectual disability. Over time, the number of beds in each unit fluctuated; at the start of the period under review, there was a second 15‐bed low secure unit for men, which closed in 2019. Not all patients on these units have been convicted of a crime, but all will have engaged in behaviour that was considered criminal.

A total of 111 patients were included in this study and demographic data are presented in Table 1. The mean age upon admission was 32.3 years. The majority of patients were male (78.4%) and 82.9% were Caucasian. The majority (93.7%) had a diagnosis of a mild intellectual disability Table 1.

**TABLE 1:** Patient demographics.

Variable	*N* (%); total = 111	Mean (SD)	Missing (%)
Age on admission (years)		32.3 (11.0)	
Sex			
Male	87 (78.4%)		
Female	24 (21.6%)		
Ethnicity			
Caucasian	92 (82.9%)		8 (7.2%)
Asian, Black, Mixed	11 (9.9%)		
Intellectual disability			
None	3 (2.7%)		1 (0.9%)
Mild	104 (93.7%)		
Moderate	2 (1.8%)		
Mild–moderate	1 (0.9%)		

### 2.2 Design and Procedure

This was a retrospective study where data were gathered about all patients admitted to Brooklands Hospital from February 2011 to March 2021. This study was registered with Coventry and Warwickshire Partnership NHS Trust as a service evaluation, and an ethical opinion from an NHS Research Ethics Committee was not required. A data capture proforma was created, and data were extracted from discharge summaries, psychology reports and electronic patient records and anonymised. The following data were captured: (1) ICD‐10 diagnosis, (2) demographic data including age, ethnicity and sex, (3) date of admission and discharge, (4) reason for admission categorised as index offence, continued care and treatment, deterioration in mental health, challenging behaviour, aggression and assessment, (5) admission source categorised as prison, community or another hospital, (6) index offence categorised as violent offences, sexual offences or arson, (7) discharge location categorised as community, low secure, medium secure or high secure, (8) a description of the treatment received while in hospital including psychological therapies and positive behavioural support (PBS), (9) length of stay, (10) neurodevelopmental disorder defined by autism and attention deficit hyperactive disorder (ADHD) diagnosis, (11) previous contact with psychiatric services including past admission, (12) previous convictions and prison sentences, (13) HCR‐20 scores and (14) the change over the admission in HONOS Scores. The HCR‐20 is a violence assessment tool that aims to predict the risk of future violence based on questions around historical and dynamic risk (Cheng et al. 2019) which was completed by the multidisciplinary team routinely at 6‐monthly intervals. The HONOS is a questionnaire that aims to measure the health and social functioning of people with intellectual disabilities (Roy et al. 2002). The aim of the tool is to measure and record progress throughout the treatment pathway and was completed by the multidisciplinary team routinely at 6‐monthly intervals. The data were collected by P.M. and R.E.

### 2.3 Data Analysis

All analyses were conducted using R statistical software (version 4.2.3—March 2023). A negative binomial regression analysis was fitted to the data using length of stay as the outcome variable. A linear regression model was provisionally fitted, but residual assumptions were violated given the skewed nature of the outcome and corresponding residual distribution; negative binomial regression provided a better fit and was used. We report Nagelkerke *R* ^2^ as a pseudo *R* ^2^ measure given that *R* ^2^ does not exist for generalised least squares. This measure indicates how well the model explains the data, but not as a measure of the proportion of variance explained. We also used a Wilcoxon signed rank test to examine whether any change on HONOS scores from admission to discharge was statistically significant.

Missingness across all variables included in the model was low at 1.3%; therefore, we did not conduct a missing data sensitivity analysis as the results should be relatively unbiased by missingness given the low proportion of missing data. An outlier was identified that indicated a single patient remained in secure services for 36,001 days, which is likely implausible and caused model fit issues; this patient was excluded from the regression model. For some patients, there was information to indicate multiple admissions, convictions and prison sentences, but the exact number of each was missing from their record. However, the available data indicated that the value was relatively high; therefore, in these instances, patient data were imputed at the 75th percentile of the variable. The HONOS and HCR‐20 scores had a high proportion of missingness (30%–40%), so we were excluded from the regression model as this significantly reduced our sample size from *N* = 110 to *N* = 58 given listwise deletion in the regression model.

## 3 Results

The average length of stay was 2053.9 days, SD = 3476.7, Table 2. The majority (76.7%) were admitted using Part III of the Mental Health Act, 1983. This means they appeared before a court in England and Wales and were ordered to hospital by a judge. The majority (89.2%) of inpatients had committed an index offence precipitating admission. During admission, 80.2% underwent some form of psychological therapy, and 69.4% had psychotropic medication prescribed upon discharge Table 3.

**TABLE 2:** Results from analyses including length of stay, ICD‐10 diagnosis, treatment received.

Variable	*N* (%); total = 111	Mean (SD)	Missing (%)
Length of stay (days)		2053.9 (3476.7)	2 (1.8%)
Mood disorder and anxiety			
No	92 (82.9%)		
Yes	19 (17.1%)		
Autism spectrum disorder			
No	92 (92.9%)		
Yes	19 (17.1%)		
Attention deficit hyperactivity disorder			
No	101 (91%)		
Yes	10 (9%)		
Psychotic disorder			
No	91 (82.0%)		
Yes	20 (18.0%)		
Personality disorder			
No	80 (72.1%)		
Yes	31 (27.9%)		
Mental health act status			
Civil	26 (23.4%)		
Forensic	85 (76.6%)		
No index offence			
No	99 (89.2%)		
Yes	12 (10.8%)		
Discharge location			8 (7.2%)
Hospital	51 (45.9%)		
Community	52 (46.8%)		
Previously known to psychiatric services			1 (0.9%)
No	36 (32.4%)		
Yes	74 (66.7%)		
Number of previous convictions		4.8 (7.1)	6 (5.4%)
Psychological therapy			
No	22 (19.8%)		
Yes	89 (80.2%)		
PBS			
No	106 (95.5%)		
Yes	5 (4.5%)		
No medication on discharge			
No	77 (69.4%)		
Yes	34 (30.6%)		

**TABLE 3:** Model output from the negative binomial regression including incident rate ratios, 95% confidence intervals and *p* values.

Predictors	Incidence rate ratios	CI	*p*
(Intercept)	**334.16**	**[189.09, 604.71]**	**< 0.001**
Age on Admission	**1.02**	**[1.01, 1.03]**	**0.002**
Sex (female)	0.90	[0.60, 1.36]	0.597
Ethnicity (other)	0.76	[0.52, 1.16]	0.189
Previously known to psychiatric services	1.15	[0.87, 1.51]	0.329
Mood disorder and anxiety	0.97	[0.67, 1.43]	0.890
Autism	**0.71**	**[0.52, 0.98]**	**0.03**
Attention deficit hyperactivity disorder	**0.39**	**[0.25, 0.62]**	**< 0.001**
Psychotic disorder	1.09	[0.73, 1.64]	0.681
Personality disorder	1.05	[0.76, 1.46]	0.786
No index offence	1.12	[0.74, 1.76]	0.605
Discharge location (community setting)	1.10	[0.83, 1.45]	0.485
Receiving psychological therapy	**3.11**	**[2.16, 4.43]**	**< 0.001**
Receiving PBS	**1.90**	**[1.06, 3.74]**	**0.044**
*R* ^2^ Nagelkerke	0.624		
N observations	93		

*Note:* Bold text = *p* < 0.05.

The results of our regression model are presented in Table 3. While holding all other variables in the model constant, those who were older upon admission and received psychological therapy and/or PBS had a significantly longer length of hospital stay. For every year increase in age on the date of admission, the incident rate ratio for length of stay would be expected to increase by a factor of 1.02 (IRR = 1.02, 95% CI [1.00, 1.03], *p* = 0.009). For those who received psychological therapy, the incident rate ratio for length of stay would be expected to be three times longer (IRR = 2.97, 95% CI [2.09, 4.16], *p* = < 0.001) and for those receiving PBS this would be expected to increase by a factor of 2.11 (IRR = 2.11, 95% CI [1.20, 4.04], *p* = 0.015). Diagnoses of ASD or ADHD were associated with a shorter length of hospital stay; the incident rate ratio for length of stay decreased by a factor of 0.71 and 0.32, respectively (ASD: IRR = 0.71, 95% CI [0.52, 0.98], *p* = 0.034; ADHD: IRR = 0.39, 95% CI [0.25, 0.62]) for those with a neurodevelopmental disorder. All other predictors were not statistically significant in the model.

The Wilcoxon signed‐rank test showed that the HONOS scores upon discharge decreased significantly (*z* = −2.83, *p* < 0.005; effect size = 0.31). The median score for the HONOS scores was 31.7 (SD = 113.0) on admission compared to 17.3 (SD = 7.0) on discharge.

## 4 Discussion

The aim of this study was to examine the relationship between patient demographics, diagnoses and treatment pathways and length of stay in secure units at Brooklands Hospital. The average length of stay was 2053.9 days or 5.63 years, and most inpatients had a history of behaviour that could be considered criminal. Over time, HONOS scores improved, and our analysis revealed that those who were older when admitted and those who received psychological therapies or positive behaviour support stayed in hospital for significantly longer, while those with a diagnosis of autism or ADHD stayed in hospital for a significantly shorter period. Other studies have reported that those who are older when they are first admitted stay longer (Völlm et al. 2017; Ailey et al. 2019) possibly because they may have a more complex history of offending and mental illness prior to admission, meaning the treatment pathway may involve more prolonged or complex interventions. It may also be the case that younger patients could be more amenable to rehabilitation work.

Our finding that those with a neurodevelopmental disorder had a shorter length of stay is inconsistent with others who have reported longer hospital stays for autistic people (Ailey et al. 2019; Kokoski and Lunsky 2009; Lunsky et al. 2009; National Autistic Society 2022), with the exception of Esan et al. (2015), who reported no difference in the length of stay for those with and without autism within specialist forensic services in the United Kingdom. It is important to mention that we made use of data from a single English hospital, which was also specialist, and it may be the case that the environment and care pathways were able to effectively meet the needs of autistic inpatients. It is of note that the authors who reported a longer length of stay for this population used data from non‐specialist inpatient psychiatric services (Ailey et al. 2019; Kokoski and Lunsky 2009; Lunsky et al. 2009). We also noted a shorter length of stay for those with ADHD, which can be managed with medications; those with ADHD who responded well to medication would have been ready for step down or community discharge earlier.

Our finding that those who received psychological therapy or PBS spent longer in hospital is interesting. While it remains possible that psychological therapy and PBS directly caused an increase in length of stay, it is more likely that patients who received psychological therapy or PBS did so because they presented with increased complexity, risk and need for treatment, thereby lengthening their stay. For some, they may need to wait for periods until specialist interventions are available, for example, sexual offender treatment programmes (Large and Thomas 2011). However, clinical complexity and risk are likely to explain the increased length of stay for this group, and others have suggested that it may take longer for this population to develop insight into their risk and develop skills to manage this risk, leading to longer lengths of stay (Taylor et al. 2017). However, there is evidence that those with intellectual disabilities within a single high security hospital in England had a shorter length of stay than those without intellectual disabilities, even though there were more similarities than differences between these two groups in terms of clinical and forensic risk (Chester et al. 2018).

## 5 Limitations

The limitations of this study are: (1) the sample was taken from a single hospital in the West Midlands in England and may not be representative of the wider British population of inpatients with intellectual disabilities, (2) while our data spanned 10 years, nevertheless, the sample size was small. This limited the number of predictor variables that we could include in our model, and we were unable to also include both HONOS and HCR‐20 scores due to the amount of missing data. Scores on the HONOS decreased over time, but how this related to length of stay could not be considered robustly, (3) data were captured from paper records and (4) length of stay is not a measure of the quality of care. A longer length of stay may be related to clinical severity (Wolff et al. 2015); this is not always the case, as other factors (e.g., lack of community services, difficulties with accommodation) may also increase length of stay (Saeed et al. 2003; Zhang et al. 2011). Every attempt was taken to ensure the accuracy of the data extracted, but some records may have been missing or may have been inaccurate. Further, we were unable to ascertain data about some social factors which may have affected length of stay (e.g., availability of housing in the community), certain psychiatric diagnoses (e.g., post‐traumatic stress disorder), different types of psychological therapies offered, nor did we capture data about Care (Education) and Treatment Reviews which have previously been shown to relate to both admission and discharge rates nationally (Langdon et al. 2023).

## 6 Implications

This study identified that patients who are older upon admission to secure units at Brooklands Hospital are more likely to remain in hospital longer. This group may be more complex and therefore requires intensive input and therapies and more complex packages of care to promote and maintain discharge. Offering effective interventions to younger patients early may help prevent future hospital admissions and promote rehabilitation.

Our finding that psychological therapies and PBS are associated with a longer length of stay is important. While it is unlikely that psychological therapies and PBS are causing a longer length of stay, Tapp et al. (2023) reported within their meta‐analysis that there is limited evidence to support the conclusion that psychological therapies are effective when used with people with intellectual disabilities; the main reason for this was due to the poor methodological quality of the included studies. A lack of evidence is not the same as evidence of a lack of efficacy, and this is markedly problematic. Robust clinical trials are needed to generate evidence about the efficacy of psychological therapies to ensure that we are offering treatments which improve mental health, recidivism and risk amongst people with intellectual disabilities. The evidence base about the use of psychological therapies and interventions within inpatient forensic services with those who do not have intellectual disabilities is also problematic and most effect sizes are small (McIntosh et al. 2021). For those with intellectual disabilities, the provision of effective psychological interventions may help promote timely discharge and a shorter length of stay. This should be a priority for future research.

## Author Contributions

**Penelope McKenna:** conceptualization, writing – original draft preparation, writing – review and editing; **Rosie England:** writing – review and editing. **Carmen Fadzelmulla‐Moreno:** writing – original draft preparation, statistical analysis. **Paul A. Thompson:** writing – original draft preparation, statistical analysis, supervision, writing – review and editing. **Harm Boer:** conceptualization, writing – review and editing. **Peter E. Langdon:** conceptualization, writing – original draft preparation, writing – review and editing, statistical analysis, supervision.

## Data Availability

The data that support the findings of this study are available from the corresponding author upon reasonable request.
